# Tips and tricks in the management of inflatable penile prosthesis infection: A review

**DOI:** 10.1080/2090598X.2021.1946335

**Published:** 2021-07-05

**Authors:** Scott C. Brimley, Ayad Yousif, Joseph Kim, Wayne J.G. Hellstrom

**Affiliations:** Department of Urology, Tulane University, New Orleans, LA, USA

**Keywords:** Prosthesis, implant, infection, salvage, erectile function, risk factors

## Abstract

**Objective:**

To review the management of inflatable penile prosthesis (IPP) infection.

**Methods**: The ‘gold-standard’ treatment for medication-refractory erectile dysfunction is the IPP, wherein the most dreaded complication is infection. To prevent and manage an infected IPP requires a strict protocol during the pre-, intra-, and postoperative course. A variety of techniques and antibiotics are used in conjunction with IPP implantation to prevent contamination. This modified Preferred Reporting Items for Systematic Reviews and Meta-Analyses (PRISMA) review of the literature examines the current practices by leading urologists in the management of IPP infection, as well as provides insights for improved patient outcomes.

**Results:**

: Patient selection is important to reduce IPP infections, and those with risk factors need to be optimised prior to surgery. Proper antibiotic prophylaxis includes pre-, intra-, and postoperative administration. As most infections derive from normal skin flora, every measure must be taken to sterilise the skin and avoid direct device skin contact. Up to 3% of virgin IPPs develop infections and this number increases to 18% in revision cases. Antibiotic coverage depends on the presenting microbe, which can vary significantly between patients.

**Conclusions:**

: A greater success in IPP implantation can be attributed to appropriate prophylaxis, field sterilisation, and surgical technique. For those implants that do become infected, often erectile function can be preserved by immediate antibiotic coverage combined with salvage procedures.

## Introduction

In 2011, 4% of all USA inpatients in acute care hospitals had at least one healthcare-associated infection, totalling 721800 hospital-acquired infections. Of these infections, 25% were associated with various implantable devices [1]. An inflatable penile prosthesis (IPP) is a permanent device with a closed-loop flow system traversing the scrotum, penis, and lower abdomen. Generally, any foreign body leads to increased risk of infection, and any early signs of device compromise should be regarded seriously due to the devastating consequences of an untreated infected device. IPP infections are relatively uncommon, but they have serious physical and psychological sequelae [1,2,3]. Strict measures should be taken to prevent infection before, during, and after the procedure. Salvage therapy is possible in some cases, depending on the clinical scenario. Here we review the proper measures and techniques that should be utilised to prevent and treat IPP infections.

## Methods

**Figure 1.**
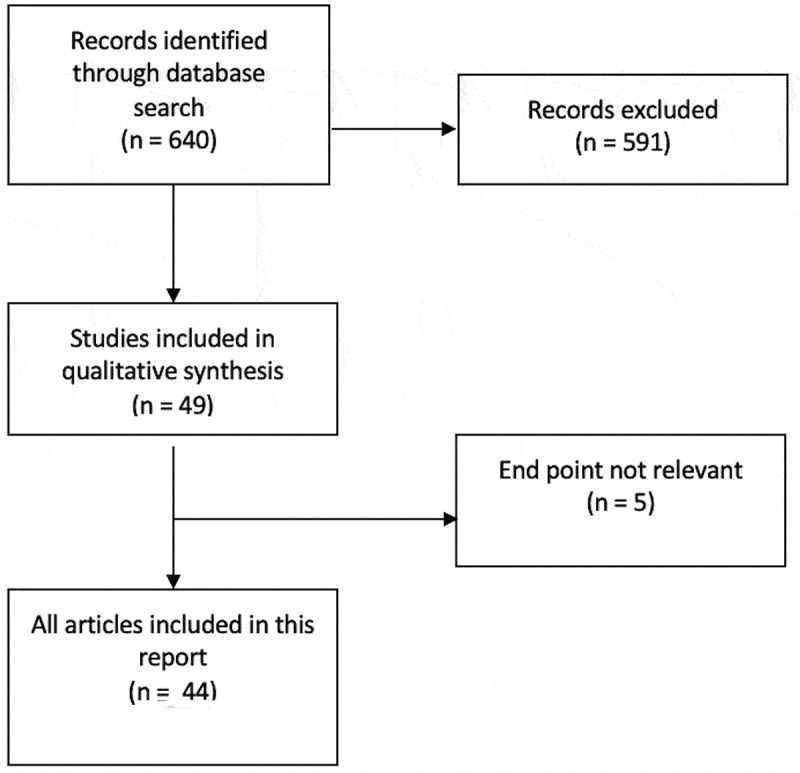
PRISMA flow chart outlines search process

A literature search was performed through the PubMed database and relevant articles were identified. Inclusion criteria are outlined in the Preferred Reporting Items for Systematic Reviews and Meta-Analyses (PRISMA) flow chart presented (Figure 1). A PubMed search of English-language articles was conducted using three separate search terms, namely ‘penile prosthesis infection’, ‘infected penile prosthesis’ and ‘infection in penile implant’. Abstracts not including the term ‘penile prosthesis infection’ were excluded

A total of 640 articles were retrieved from PubMed. In all, 591 were excluded and 49 abstracts screened and read of which five were excluded, as their end point was not relevant to IPP infection; thus, 44 were included in the present review (Figure 1)

## Preoperative measures

### Risk factors and patient selection

An IPP is indicated for patients who have failed first- and second-line therapies for erectile dysfunction (ED; phosphodiesterase-5 inhibitors and intracavernosal injections, respectively). IPPs may also be offered to patients with contraindications to these therapies, or to patients with an aversion to needles. Clinicians must conduct a thorough history to ascertain which risk factors the patient has in terms of infection, early erosion, and psychological dissatisfaction. Patients without a complete understanding of the details of an IPP may be disappointed with the results.

In a 2011 study of IPP revision, the most common risk factor was diabetes mellitus (DM) [3,4]. This study recorded that the DM population had a 1.72% revision rate vs 1.26% in the non-DM population. It has been suggested that haemoglobin A1c values can be a predictor of infection rates, but there has not been consistent evidence to support this [3,5,6]. Yet, the theoretical risk is high; thus, some urologists choose a specific cut-off haemoglobin A1c level at which they will not perform the surgery [7].

Substance abuse has been established as a risk factor for infection of orthopaedic implants, as it is associated with bacteraemia following needle injection [8]. A 2016 article published in the *Journal of Sexual Medicine* documented a 30–40% increase in postoperative infections in polysubstance abusers undergoing IPP placement, as compared to patients who were not polysubstance abusers at the time of surgery [9]. This same study also found that homelessness was a risk factor for postoperative prosthetic infection.

An immunocompromised state is an obvious risk factor for infection, and any HIV-positive patient should have an optimised cluster of differentiation 4 (CD4) count prior to surgery. Other risk factors include spinal cord injuries, previous IPP placement, and active infection elsewhere [10].

### Laboratory assessments and patient instructions

Generally, it is provider preference to obtain preoperative urine culture, and 60% and 50% of Sexual Medicine Society of North America (SMSNA) and International Society for Sexual Medicine (ISSM) members, respectively, continue to do so [11]. After the appropriate patient has been selected for surgery, with all risk factors optimised, the patient must have routine urine analysis and culture [12]. During the surgery, a Foley catheter is placed to drain the bladder and assist the surgeon in identification of the urethra. The catheter usually remains overnight to allow for proper bladder drainage, while on analgesics. Any active infection must be appropriately treated prior to the day of surgery, regardless of symptoms. It has been theorised that the Foley catheter can be a source of bacteraemia and compromise the IPP outcome, although most infections of these devices are attributable to normal skin flora.

### Antibiotic prophylaxis

The evidence for prophylactic antibiotic coverage for an IPP is less than desired, although it is well-established for other surgeries involving foreign bodies [13]. On this basis, multiple review articles support the use of oral antibiotics (either sulfamethoxazole/trimethoprim or ciprofloxacin) 2 days prior to surgery [14,15]. The steps for preoperative management are included in Table 1 [14].

**Table 1.** Checklist for preoperative antimicrobial management [14]

Preoperative	Urine culture
	Optimise haemoglobin A1c (≤7%)
2 days prior to surgery	Oral antibiotics
	Hibiclens® scrub twice a day
Day of surgery	Wash genitals with soapy water
	Antibiotics: gentamycin and vancomycin (second-generation cephalosporin)
	Remove hair with clippers
	Chlorhexidine scrub prep

## Intraoperative measures

### Microbiology

Typically, an infected IPP occurs through exposure to normal skin flora, indicating contamination during the actual implantation procedure. These bacteria are generally coagulase negative staphylococcus (e.g. *Staphylococcus epidermidis*) and the infections are typically less virulent, more indolent in course, and occur after a longer interval of time has passed since implantation. Signs are non-specific and include new-onset pain, warmth, erythema, and adhesions of the pump to the scrotal skin. Such infections need to be treated regardless of negative cultures, normal white counts, or lack of systemic signs, as the infection will likely progress with increasing pain that leads to the inability to use the device. The formation of biofilms is a main mechanism by which these types of prosthetic infections evade immunodetection.

Infections of IPPs within 2 months of surgery that appear more severe, and are associated with toxicity, elevated white blood count, and systemic signs are likely due to more virulent bacteria (e.g. *Staphylococcus aureus, Klebsiella, Serratia, E. coli, Pseudomonas*).

### Antibiotic prophylaxis

Since the first successful implantation of an IPP was published in 1973, advances in their mechanical structure and function have steadily led to increased survival time of the devices [16]. Before prostheses were coated with antibiotics, infection rates in the first cases were reported as high as 3–10% [17]. A long-term survival analysis of re-implant infection rate for the AMS-700™ (Boston Scientific, Marlborough, MA, USA) prosthesis with minocycline and rifampicin demonstrated that only 2.5% developed infections after an average 6.6-year follow-up. Without antibiotic-coated devices, the infection rate was recorded at a mean of 3.7% [18]. Similar results were reported with the Coloplast (Coloplast, Minneapolis, MN, USA) device [19], although there is evidence that the Coloplast’s hydrophilic coating produces substantial antimicrobial properties in addition to the antibiotic solution the device is dipped in (rifampicin-gentamicin or vancomycin-gentamicin) [20]. A 2020 study compared the impact of different dipping solutions on postoperative infection [21]. The authors observed that the combination of vancomycin and gentamycin dipping solution resulted in the lowest infection rate, while rifampicin inclusion increased the infection rate, possibly indicating a change in microbial resistance patterns.

Perioperative antibiotic prophylaxis is recommended for most surgical procedures, but the choice of coverage varies greatly depending on the type of surgery, provider, and location. The AUA Best Practice Guidelines recommend using an aminoglycoside (if renal insufficient: aztreonam) plus vancomycin or a first- or second-generation cephalosporin.

### Sterilisation

Standard surgical technique includes perioperative hair removal in the operating room prior to sterilisation. There is a trend toward clippers due to their reduction of infection rate in most other surgeries [22]. However, razors are often used over clippers, as they cause less skin abrasions due to the genital skin’s elasticity and thinness [23].

As most infections occur from skin flora, surgical site antisepsis is a vital part of infection prophylaxis. Currently, there is level 1 evidence in favour of chlorhexidine and alcohol over betadine for non-mucosal surfaces [24]. However, there are limited data on the infection difference for IPP surgeries without any prospective trial showing significant difference in infection rate, although positive cultures were found to be higher in the betadine group [25].

### Techniques

Poor surgical technique and prolonged surgeries are recognised sources of increased infection. Multiple studies have shown the benefits regarding infection reduction when adhering to a checklist [10]. It is often the surgeon, not the patient, that is to blame for prosthetic infections. The ‘no-touch’ technique demonstrated that less contact with the patient’s skin, the fewer infections occurred. With the addition of coated-IPP devices and the no-touch technique, the infection rate has been reported as 0.46% [20]. Throughout the implant procedure, the surgeon and assistant should avoid contact with the patient’s skin and consider changing gloves during different steps of the procedure.

## Postoperative measures

Infection of an IPP is a dreaded complication for both physician and patient, with serious physical and emotional morbidities for patients. It has a reported incidence of 1–3% in naïve cases and up to 18% in revision cases [26].

Most device infection is due to device contamination at the time of implantation. *S. epidermidis*, a common skin flora, is the primary culprit implicated in device infection. The introduction of prosthetic coating is gradually changing the microbes found in infected IPPs, trending toward more systemic responses and purulent wounds [3]. Gross et al. [27] conducted a multi-institutional study from 25 centres that obtained cultures at the time of explantation or salvage. The study revealed 73% and 39% of cultures were positive for gram-positive and -negative bacteria, respectively. *S. aureus* was encountered in nearly a third of positive cultures and *Candida* species was present in 11.1% of positive cultures. Anaerobes were found in 10.5% of cultures. Men with factors that increase the risk of infection should be treated with broad-spectrum antibiotics and antifungals [28].

Increased risk of device infection has been noted to be higher in certain patient populations, such as immunosuppressed patients and patients with DM, those with spinal cord injury and revision procedures [29]. Additionally, prolonged hospitalisation increases the risk of implant infection due to change of skin flora to more virulent types [30].

The manifestations of device infection can be subclinical or clinically evident (acute). A reported 56% of IPP infections occur within the first 7 months after surgery, 36% from 7–12 months, and 2.5% after 5 years [31]. Patients with subclinical infections often present with chronic device discomfort with no obvious systemic symptoms. A trial of prolonged course of oral antibiotics for 10–12 weeks has been suggested for this subset of patients and, for those who fail to show any improvement with antibiotics, surgical intervention is the next step [30]. In this author’s experience, these patients may respond to intravenous vancomycin weekly for a couple of weeks. If the discomfort abates for a short interval, this is suggestive of a subclinical infection (*S. epidermidis*) and the need for a surgical salvage procedure [32]. If a patient presents with obvious signs of infection such as fever, penile pain, purulent discharge, or extrusion of any of the device components, then explantation of the entire device is usually recommended.

In patients with clinically evident implant infection, the traditional treatment consists of removal of the entire device components, as well as any other associated foreign material. Perioperative antibiotics should be administered, wound cultures are obtained, and direct wound irrigation is performed. However, the approach of explantation and waiting for 3–6 months before re-implanting is associated with significant corporal fibrosis and penile shortening, making subsequent penile implantation more challenging, with lower patient satisfaction.

As a modification of the above complete removal approach, Mulcahy [33] suggested intracorporal irrigation and drainage with antibiotics solution for 3 days, using drains placed in the proximal and distal cylinder compartments. The goal of this technique is to eradicate infection and minimise corporal fibrosis, thus facilitating subsequent device implantation. New implants can be inserted several months later and, in the interim, use of a vacuum device is recommended to limit penile shortening and curvature.

Brant et al. [34] introduced the salvage technique in 1996, which involves removal of all components of the device with thorough wash-out of the infected area (corpora, pelvis, and scrotum). This protocol involves seven steps of irrigation starting with kanamycin (80 mg/L) and bacitracin (1 g/L) in normal saline, by half-strength hydrogen peroxide and half-strength povidone-iodine solution, 5 L normal saline containing vancomycin (1 g) and gentamycin (80 mg) for pressurised irrigation. This is followed by a change of drapes, instruments, gowns, and gloves, and a new implant is inserted. The initial series included 11 patients with a success rate of 91%, while it was 83% in the following series that included 55 patients [34,35]. The favourable results of this salvage technique were reproduced by other studies conducted by Knoll [36] and which showed an infection-free rate of 80% and 86%, respectively.

To assess the infection outcome after malleable salvage technique and to evaluate the utility of subsequent conversion to an IPP, a multicentre study by Gross et al. [37] demonstrated an infection-free rate of 93% (54 of 58 patients), with a postoperative mean (range) follow-up of 8.4 (1–84) months. Of note, mechanical irrigation is required to break the biofilm that harbours the bacteria. Biofilm consists of bacteria enclosed in a self-made extracellular polysaccharide matrix. Bacteria within biofilm can tolerate and survive pH changes, antibiotics, phagocytosis, and nutrient deprivation. Contraindications to salvage procedures are cylinder extrusion, severe sepsis, DM with severe ketoacidosis, significant tissue necrosis and rapidly developing infections [30].

Swords et al. [38] suggested the use of intracorporal antibiotic casts for infected penile implants in patients who are not candidates for the salvage procedure. High purity CaSO_4_, a compound used in the field of orthopaedics as a bone void filler, is mixed with tobramycin and vancomycin to create a paste that is injected into the corporal space. The cast will mould after closure of the corpora and acts as filler, preventing corporal fibrosis and preserving penile length. Also, it is hypot3hesised that the cast reduces the risk of infection due to prolonged tissue exposure to antibiotics. The cast dissolves in 4–6 weeks and subsequent penile implantation is easier, as there is minimal-to-no corporal fibrosis. Table 2 summarises the outcomes of various retrospective studies regarding the possible management options for infected IPP.

**Table 2.** Possible treatment options for infected IPP

Reference	Technique	Year of publication	Study	Success rate, *n/N* (%)
Brant et al. [34]	Immediate salvage	1996	Retrospective	10/11
Knoll et al. [36]	Immediate salvage	1998	Retrospective	8/10
Knoll et al. [36]	Delayed salvage	1998	Retrospective	22/31 (71)
Kaufman et al. [39]	Immediate salvage	1998	Case reports	6/7
Mulcahy [35]	Immediate salvage	2000	Retrospective	45/55 (82)
Gross et al. [37]	Malleable salvage	2016	Retrospective	54/58 (93)

### Postoperative antibiotics

Another common practice by implanting surgeons is the prescription of postoperative antibiotics. Wosnitzer and Greenfield [39] conducted a survey regarding postoperative use of antibiotics among urologists, which included 52 members of the SMSNA and 164 non-SMSNA members. The study showed that 94% and 88% of the SMSNA and non-SMSNA urologists prescribe postoperative oral antibiotics, respectively.

The AUA Best Practice Statement on Urologic Procedures and Antibiotic Prophylaxis (2019) recommends that antibiotics administration should be limited to the duration of surgery and no antimicrobial should be continued postoperatively. However, the European Association of Urology (EAU) recommends postoperative antibiotics for >24 h to reduce the risk of IPP infection, although it lacks supporting evidence [40,41].

Two recent studies examined the association between penile prosthesis infection and administration of postoperative antibiotics. The first was by Adamsky et al. [42] and included 10,847 patients between 2003 and 2014. Antibiotics were prescribed in 6578 patients, with 4269 that were not. The device was explanted in 148 patients who received oral antibiotics and in 80 patients who did not (antibiotics vs no antibiotics IPP: 2.2% vs 1.9%, *P* = 0.18). The study reported that DM and IPP revision surgery was associated with increased risk of explantation, while postoperative antibiotics did not reduce that risk.

The other study was a retrospective review by Dropkin et al. [43,44]. It included 222 patients who presented for IPP insertion and were divided into three groups. The 88 patients in Group 1 had no risk factors for infection and did not receive postoperative antibiotics; Group 2 patients included 48 patients who had risk factors for infection and received no postoperative antibiotics; and 86 patients in Group 3 patients had risk factors for infectious complications and received postoperative antibiotics. The risk factors included a history of DM, immunosuppression, active smoking, previous IPP insertion, and spinal cord injury. No statistical difference was encountered among the groups in device explantation due to infectious complications (0% vs 4% vs 5%, *P* = 0.130). The authors concluded that patients undergoing IPP insertion are unlikely to benefit from routine administration of postoperative antibiotics.

## Conclusion

The insertion of an IPP is the ‘gold-standard’ treatment of choice for medication-refractory ED. These devices can significantly improve the quality of life for both men and their partners. Nevertheless, infection can still occur, as foreign bodies are a nidus for infection. The present systematic review of the literature regarding infected IPPs has shown the importance of preoperative patient selection and guidance, intraoperative technique, and postoperative management. Bacteria that are introduced at the time of surgery may adhere to the prosthesis and evade antibiotics by forming biofilms. Additionally, the reduced blood flow to the cavernosal tissues in many of these patients with ED with arterial insufficiency provides inadequate antibiotic delivery to the cavernosal tissues. Prosthetic infection causes significant physical, emotional, and psychological distress to these patients. Proper patient selection is an obvious key to reducing the rate of infection. For those patients who do eventually undergo the prosthetic surgery, proper medical optimisation, antibiotic prophylaxis, and surgical technique are paramount. Further controlled studies will help us better understand patient risk factors with the aim to continue to improve patient outcomes.
